# Evolution of Middle Ear Modelling Techniques: A Review

**DOI:** 10.7759/cureus.20829

**Published:** 2021-12-30

**Authors:** Sana Parveen, Shraddha Jain, Sunil Kumar, Sourya Acharya, Dhruv Talwar

**Affiliations:** 1 Department of Otolaryngology, Jawaharlal Nehru Medical College, Datta Meghe Institute of Medical Sciences (Deemed to be University), Wardha, IND; 2 Department of Otorhinolaryngology, Jawaharlal Nehru Medical College, Datta Meghe Institute of Medical Sciences (Deemed to be University), Wardha, IND; 3 Department of Medicine, Jawaharlal Nehru Medical College, Datta Meghe Institute of Medical Sciences (Deemed to be University), Wardha, IND

**Keywords:** ct temporal bone, stapes, incus, malleus, middle ear

## Abstract

This review article attempts to analyze the various research studies conducted in developing the models to evaluate the anatomy of the middle ear, its biomechanics, and the applications of these models in normal and diseased states.

Various studies conducted over the past 50-60 years have been critically analyzed. We also discuss the various advantages and disadvantages of different methods of measurement of middle ear parameters. Beginning from anatomical modelling to histopathological sections and the latest three-dimensional (3D) reconstruction with finite element modelling, various methods of middle ear measurements have been critically analyzed.

At the end of this review, we have concluded that the best and most effective method of middle ear modelling is the 3D reconstruction using high-resolution computed tomography and finite element modelling.

## Introduction and background

The middle ear is a rather complex structure that largely influences hearing, disease processes, and reconstruction of hearing mechanisms [1]; hence, understanding the middle ear physiology is important. Various middle ear models have been developed to assess acoustics of the middle ear, and continuous refinements have been done in their design over years. Initially, static models were developed to understand the physiology of hearing and the role of the middle ear in amplification. However, these had limitations in terms of reproducibility of results in live subjects, as middle ear structures are affected by stress, strain, and differences in the thickness of the tympanic membrane at various sites.

Surgical techniques and materials have been constantly revised by the surgeons, based on their experience and existing knowledge of physiology. However, the knowledge of middle ear physiology and biomechanics is based mainly on the experiments conducted by engineers, who may not be aware of the medical challenges. Therefore, an otolaryngologist’s opinion on this issue was highly warranted; hence, the current review was undertaken to discuss the evolution of middle ear modelling techniques and their role in studying the biomechanics of the middle ear, in normal and diseased states.

Search strategy

**Figure 1:**
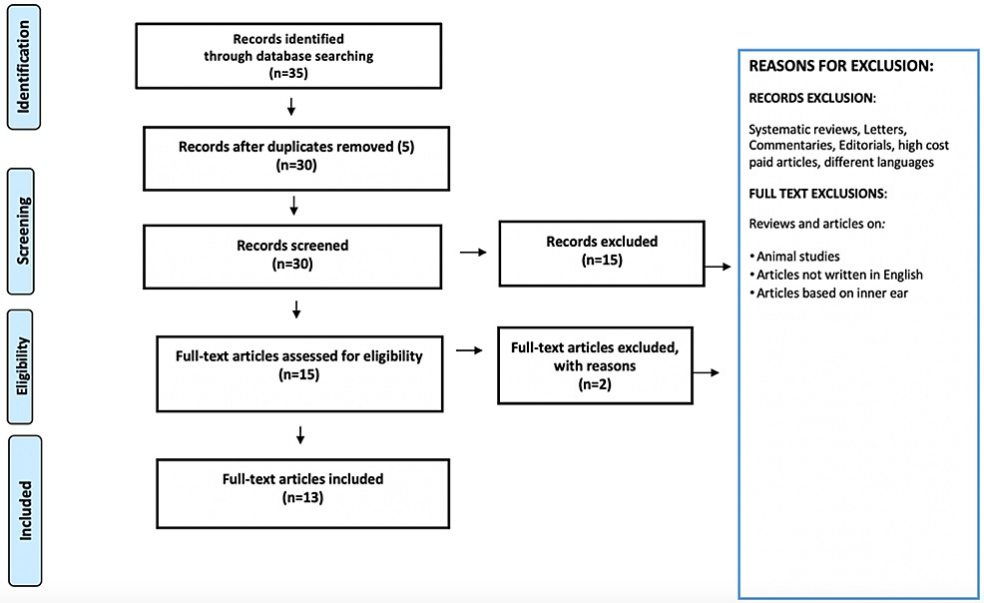
Search strategy of the article.

The present review was performed addressing the Preferred Reporting Items for Systematic Reviews and Meta-Analyses (PRISMA) guidelines (Figure 1) to assess the literature based on the evolution of middle ear modelling techniques and their clinical applications. Manual and electronic data resources were accessed and articles published before December 2021 were included for this review. The following databases were used in searching the literature: PubMed, Scopus, Web of Science, and Google Scholar. The articles written in English were included in this review. The words used for search strategy were: "middle ear model" {Medical Subject Heading terms} OR "Malleus" OR "Incus" OR "Stapes" AND "Computed Tomography".

Selection criteria

The selection criteria for the present review included randomized as well as non-randomized controlled trials, cohort studies, and prospective studies based on live humans as well as cadaveric temporal bones along with retrospective radiological studies. Technical reports, animal studies, letters to the editor, and case reports were excluded from the present review.

## Review

Middle ear modelling

Middle ear modelling comprises two main steps: reproducing the dimensions of the middle ear cavity and making the model.

Reproducing the Dimensions of the Middle Ear Cavity

Reproducing the dimensions of the middle ear cavity requires static and dynamic measurements. Static measurements include dimensions of middle ear cavity, tympanic membrane, and ear ossicles by various methods as depicted in Table 1. These are known as boundary conditions.

**Table 1:** Various static methods of middle ear measurements. HRCT, high-resolution computed tomography.

Static methods of measurements of the middle ear cavity	Technique	Advantages	Disadvantages
Cadaveric bones physical measurements [2]	Serial bony sections are achieved with a real microtome.	Maximum detail, extremely precise, and accurate.	Extremely cumbersome and requires tonnes of experience and skill. Embedding and decalcification of bony specimen prior to mounting of slices on an electron microscope and sectioning on plates of glass, followed by staining.
HRCT imaging of normal ear [3]	Series of serial sections derived by virtual or physical sectioning using CT.	Helps in identifying the structures of interest and can help in the making of three-dimensional models of the middle ear.	More useful for bony elements, not for identifying nerves.
X-ray micro-CT [4]	Virtual sections of middle ear resolutions found. The thickness of the bone around the middle-ear ossicles needs to be minimized so as to best visualize the middle ear bones because it absorbs the most amount of radiation.	An accurate model of the ossicular chain of the human ear can be constructed.	Requires prior knowledge of the shape, density of the middle-ear ossicles.
MRI temporal bones [5]	The middle ear cleft and cavity are filled with contrast dye that has a high absorptive capacity of magnetic resonance and then the ossicles will light up as islands within a fluid-filled cavity.	The definition can be compared to that of X-ray micro-CT.	Care has to be taken not to introduce any bubbles of air. Around five to 10 times more costly than an X-ray scanning machine for this purpose [6].

Dynamic measurements include the measurement of the velocity of the tympanic membrane, which is measured by laser Doppler vibrometer, stress, strain, and measurements of immittance of the ossicles and tympanic membrane, as depicted in Table 2. These are known as loading conditions.

**Table 2:** Various methods of recording dynamic parameters of the middle ear.

Method	Description	Advantage	Disadvantage
Laser Doppler vibrometer [6]	Promptly and accurately measures the velocity of the tympanic membrane induced by sound, near umbo (malleus inferior tip) among patients and living human subjects.	Selective and sensitive method of differentiation and diagnosis of numerous ossicular disorders in diseased individuals having intact tympanum and well-aerated middle ear cavities. Can differentiate between the middle ear and inner ear pathologies.	Requires high-quality software, which is expensive and not easily available everywhere.
Live functional measurements [7]	Measurements of the immittance [8] of middle ear input done in vitro, in temporal bones obtained from human cadavers are contrasted against parallel parametric measurements from normal subjects, in vivo.	Results were comparable in both cadaveric and human temporal bone.	Dried up temporal bones can give erroneous results. The difference in static pressures on each side of the eardrum should be the same.

Material data of each part are shown in Table 3. These data are determined by referring to the research of Higashimachi et al. [4] and Koike et al. [9] to give a combined analysis of Young’s modulus, density, and Poisson’s ratio of the tympanic membrane, ossicles, and the ligaments.

**Table 3:** Young’s modulus, density, and Poisson’s ratio of various middle ear components.

Anatomical landmark	Young’s modulus (MPa)	Density (kg/m^3^)	Poisson’s ratio
Tympanic membrane	33.4	1,200	0.3
Malleus, incus, stapes	13,436	4,350	
Lateral, superior, and anterior mallear ligament	21	2,500	
Superior and posterior incudal ligament and stapedial annular ligament	0.65	2,500	
Incudostapedial and incudomalleolar joint	6	1,200	
Stapedial muscle	0.52	2,500	
Base-plate	1 x 10^10 ^	-	

The malleus, incus, and stapes had similar Young’s modulus and density, whereas the tympanic membrane was found to be less dense. These parameters were comparable for lateral, superior, and anterior malleolar ligaments; however, these values were much lower for the anterior and superior incudal and stapedial annular ligaments.

Making a Middle Ear Model

Static modelling: Various methods have been used by scholars to create static models of the middle ear, beginning from serial histological sections from cadaveric temporal bones to three-dimensional (3D) modelling from CT images, as shown in Table 4. These can be used to make dedicated geometrical models for their utilization in middle-ear biomechanical research studies [10].

**Table 4:** Various types of static middle ear modelling methods. 3D, three-dimensional.

Types of static modelling	Technique	Advantages	Disadvantages
Physical model [10]	The full-size physical model is an artificial representation of the tympanic membrane, the auditory ossicles, and three ligaments. The structure of the model is made up of silicone for bony parts and silicone sheets for the tympanic membrane.	Reproduces the basic characteristics of a real human middle ear relatively accurately, also in terms of 3D effects[10].	Immobile model, cannot reproduce movements of the tympanic membrane and ossicular chain.
3D model [11]	The mastoid X-rays are compared with temporal bone CT scans by synchrotron radiation, using fluorescence optical sectioning, magnetic resonance microscopy, and physical serial sections, and a 3D middle ear model is constructed [4].	Used in the making of design of mathematical models of parts of the ear and teaching models.	Requires expensive software for conversion of CT and MRI images into 3D images.

Dynamic modelling: The creation of a dynamic model needs the incorporation of some extra parameters for the creation of the middle ear model. These include vibratory properties of the tympanic membrane, stress, and strain of ossicles and TM, measured by Young’s modulus (Table 5).

**Table 5:** Various types of dynamic middle ear modelling methods.

Types of dynamic modelling	Technique	Advantages	Disadvantages
Finite element model [12]	The object of interest is divided into numerous small simplified mesh elements. The applied forces and mechanical properties are depicted by functions defined over each and every element known as mesh particles and the mechanical response of the system as a whole is computed.	Takes into account the phase-shift moiré shape dimensions to accurately define the shape of the tympanic membrane.	Requires expensive software for conversion of CT images into finite element model.
Acoustic modelling [13]	Formulation of a circuited lumped-element model of the adult middle-ear of human beings for biomechanics, according to the comparisons taken from measuring air-conduction information.	Incorporates the acoustic effects of the middle ear cavity, antrum, and aditus, as well as third-window effects, which are not included in any of the previously described models.	Requires highly skilled professional assistance for the development and analysis for working of the model.

## Conclusions

Through this review article, we have attempted to analyze the different methods of middle ear modelling, which have been done in the form of physical, acoustic, finite element modelling, and 3D reconstruction using X-ray micro-CT.

These can be converted into finite element models for clinical applications to assess the properties in normal ears as well as to assess the status of the tympanum and ossicles and middle ear volume in diseased conditions.

It is also important to identify which method of modelling is the best to assess the hearing gain/loss after the ear surgeries. The best method would be a combination of 3D reconstruction with finite element modelling using high-resolution computed tomography scans of the temporal bones, for analysis of the ossicular chain and its various properties.
